# Evaluation of an international educational programme for health care professionals on best practice in the management of a perinatal death: IMproving Perinatal mortality Review and Outcomes Via Education (IMPROVE)

**DOI:** 10.1186/s12884-016-1173-8

**Published:** 2016-11-25

**Authors:** Paul A. Gardiner, Alison L. Kent, Viviana Rodriguez, Aleena M. Wojcieszek, David Ellwood, Adrienne Gordon, Patricia A. Wilson, Diana M. Bond, Adrian Charles, Susan Arbuckle, Glenn J. Gardener, Jeremy J. Oats, Jan Jaap Erwich, Fleurisca J. Korteweg, T. H. Nguyen Duc, Susannah Hopkins Leisher, Kamal Kishore, Robert M. Silver, Alexander E. Heazell, Claire Storey, Vicki Flenady

**Affiliations:** 1 Mater Research Institute, The University of Queensland, Level 2 Aubigny Place, South Brisbane, QLD 4101 Australia; 2 International Stillbirth Alliance, Bristol, UK; 3 Perinatal Society of Australia and New Zealand Stillbirth and Neonatal Death Alliance, Monington, Australia; 4 Medical School, Australian National University, Canberra, Australia; 5 Centenary Hospital for Women and Children, Canberra, Australia; 6 School of Medicine, Griffith University, Brisbane, Australia; 7 Gold Coast University Hospital, Southport, Australia; 8 Charles Perkins Centre, The University of Sydney, Sydney, Australia; 9 Newborn Care, RPA Women and Babies, Royal Prince Alfred Hospital, Sydney, Australia; 10 Mater Health Services, Brisbane, Australia; 11 Kolling Institute, The University of Sydney, Sydney, Australia; 12 Children’s Hospital at Westmead, Sydney, Australia; 13 Melbourne School of Population and Global Health, University of Melbourne, Melbourne, Australia; 14 University of Groningen, Groningen, The Netherlands; 15 Department of Obstetrics and Gynecology, Martini Hospital, Groningen, The Netherlands; 16 Institute for Reproductive and Family Health, Hanoi, Vietnam; 17 College of Medicine Nursing and Health Sciences, Fiji National University, Suva, Fiji; 18 Health Services Center, University of Utah, Salt Lake City, USA; 19 Maternal and Fetal Health Research Centre, University of Manchester, Manchester Academic Health Science Centre, Manchester, UK

**Keywords:** Stillbirth, Neonatal death, Perinatal death, Clinical practice, Education, Training

## Abstract

**Background:**

Stillbirths and neonatal deaths are devastating events for both parents and clinicians and are global public health concerns. Careful clinical management after these deaths is required, including appropriate investigation and assessment to determine cause (s) to prevent future losses, and to improve bereavement care for families. An educational programme for health care professionals working in maternal and child health has been designed to address these needs according to the Perinatal Society of Australia and New Zealand Guideline for Perinatal Mortality: IMproving Perinatal mortality Review and Outcomes Via Education (IMPROVE). The programme has a major focus on stillbirth and is delivered as six interactive skills-based stations. We aimed to determine participants’ pre- and post-programme knowledge of and confidence in the management of perinatal deaths, along with satisfaction with the programme. We also aimed to determine suitability for international use.

**Methods:**

The IMPROVE programme was delivered to health professionals in maternity hospitals in all seven Australian states and territories and modified for use internationally with piloting in Vietnam, Fiji, and the Netherlands (with the assistance of the International Stillbirth Alliance, ISA). Modifications were made to programme materials in consultation with local teams and included translation for the Vietnam programme. Participants completed pre- and post-programme evaluation questionnaires on knowledge and confidence on six key components of perinatal death management as well as a satisfaction questionnaire.

**Results:**

Over the period May 2012 to May 2015, 30 IMPROVE workshops were conducted, including 26 with 758 participants in Australia and four with 136 participants internationally. Evaluations showed a significant improvement between pre- and post-programme knowledge and confidence in all six stations and overall, and a high degree of satisfaction in all settings.

**Conclusions:**

The IMPROVE programme has been well received in Australia and in three different international settings and is now being made available through ISA. Future research is required to determine whether the immediate improvements in knowledge are sustained with less causes of death being classified as unknown, changes in clinical practice and improvement in parents’ experiences with care. The suitability for this programme in low-income countries also needs to be established.

## Background

The loss of a child as a stillbirth or neonatal death is a tragedy which is often associated with long term adverse outcomes for parents, families and the health care provider [1, 2]. In addition to providing compassionate care, determining the causes of perinatal death is an essential part of quality assessment. The purpose of investigations from the family’s perspective is to provide an explanation for the death, to enable appropriate counselling about recurrence risk, and to inform the management of future pregnancies. From the health system perspective, investigation of perinatal deaths provides information on the individual case, insights into avoidable systems issues, information on the overall health of the community, and can instigate changes in clinical practice and stimulate research [3–5]. From a health perspective, having accurate information can be used to inform public health initiatives.

The Perinatal and Maternal Mortality Review Committee in New Zealand reported that only 44% of perinatal deaths were optimally investigated [3]. While there is some debate about the optimal perinatal autopsy rate, [4]. guidelines for perinatal mortality from the Perinatal Society of Australia and New Zealand recommend that all parents be offered the option of a high quality autopsy following stillbirth or neonatal death [5]. However perinatal autopsy rates are low and vary considerably from 31% in Queensland [6] to 62% in Western Australia [7] reflecting the challenges faced in implementing this recommendation. While the recent guideline from the Royal College of Obstetrics and Gynecology does not suggest an optimal perinatal autopsy rate they recommend that it is offered and performed by appropriately trained pathologists [4]. There is little published on international rates of perinatal autopsy with much of the research from middle- and high-income countries with a suggestion that the rate is declining [8, 9]. A major limiting factor is the discomfort some clinicians experience in broaching this question, negative views and attitudes on the value of autopsy from clinicians and parents, cost and lack of availability in different countries [8, 9]. Providing bereavement care and counselling regarding investigations that meets the needs of parents is difficult and parents report that health care providers are frequently under equipped for this task [10].

In order to improve investigation and audit on causes of perinatal death, to inform prevention strategies, and to enhance care that parents receive around the time of a stillbirth or neonatal death, the Perinatal Mortality Group of the Perinatal Society of Australia and New Zealand (PSANZ) developed Clinical Practice Guidelines for Perinatal Mortality [5]. Despite evidence that increased use of the guidelines can reduce the proportion of stillbirths classified as unexplained, [11] there was a lack of awareness of the guidelines in both Australia and New Zealand [12].

The IMproving Perinatal Mortality Review and Outcomes Via Education (IMPROVE) programme (https://sanda.psanz.com.au/clinical-practice/improve/) was developed to increase the uptake of the PSANZ Clinical Practice Guidelines for Perinatal Mortality and has undergone constant revision since its inception based on review of latest evidence and feedback from attendees [13]. IMPROVE uses a Structured, Clinical, Objective, Referenced, Problem-orientated, Integrated and Organised (SCORPIO) [14] approach to teaching. SCORPIO is a medium for skills training based on small-group, participant centred, multi-professional teaching. To ensure the high quality clinical care in this very challenging area, the target audience for IMPROVE is the multidisciplinary team involved in the care of babies and families around the time of perinatal death with a focus on medical officers (neonatologists, obstetricians, pathologists) and midwives, but also including neonatal nurses, and allied health staff (social workers, bereavement specialists). The programme has been endorsed by the Royal Australian and New Zealand College of Obstetricians and Gynaecologists, the Australian College of Midwives and the Australian College of Neonatal Nurses, and the Victorian Consultative Council on Obstetric and Paediatric Mortality and Morbidity (COPMM). Participants attending the IMPROVE programme can obtain Continuing Practice in Education Points from the relevant organisation.

The aims of this study were to determine the effectiveness of the IMPROVE programme in terms of change in participants’ knowledge of and confidence in the management of perinatal deaths, along with satisfaction with the programme. We also aimed to determine the suitability of this programme for international use in middle and high income countries.

## Methods

### Education programme

The IMPROVE programme is delivered via workshops that consist of a short introductory lecture with a pre-programme evaluation; six learning stations; a formative assessment and a post-programme evaluation. This method adapts the PSANZ Clinical Practice Guidelines for Perinatal Mortality into six hands-on, skill-based and dynamic rotating teaching stations utilising a tell-show-do-feedback methodology (Table 1). Each teaching station is based on key recommendations of the PSANZ guidelines: perinatal mortality classification (Station 5: Audit and classification of perinatal deaths), investigation (Station 3: Investigation of perinatal deaths), autopsy consent (Station 1: Communicating with parents about perinatal autopsy), placenta and post mortem examination (Station 2: Autopsy and placental examination), examination of the baby (Station 4: Examination of babies who die in the perinatal period), and perinatal bereavement (Station 6: Psychological and social aspects of perinatal bereavement). The programme incorporates both didactic and interactive educational elements. While there is some tailoring for local practices the programme content has been kept similar across all delivery settings. While SCORPIO recommends an optimum number of six participants per small group, [14] for pragmatic reasons this number has been increased to nine for some workshops. Participants spend 30 min at each station. Participants are provided a study guide containing the aims and objectives of each station, a copy of material from the PSANZ Clinical Practice Guidelines for Perinatal Mortality that may be used in future clinical practice, e.g. checklist for clinical examination of the baby, list of perinatal mortality classifications, brochures for explaining autopsy to parents, and a list of suggested readings. All educators involved in the programme have extensive clinical experience and participate in a train-the-trainer programme to ensure compliance with the SCORPIO methodology, and also the quality and consistency of IMPROVE.

**Table 1:** Content of the improving perinatal mortality review and outcomes via education (IMPROVE) programme

Station	Aim	Objectives
Introduction	To provide an overview of: perinatal mortality in Australia with a focus on stillbirth in the international context; the Perinatal Society of Australia and New Zealand (PSANZ) Clinical Practice Guideline for Perinatal Mortality; and, the IMPROVE programme	1. Understand the basic epidemiology of perinatal mortality and stillbirth 2. Understand the scope and purpose of the PSANZ Guidelines 3. Know the definitions of perinatal mortality applied in the appropriate jurisdiction 4. Understand the rationale for the IMPROVE Programme
1. Communicating with parents about perinatal autopsy	To provide information to assist clinicians to help parents to make an informed choice about perinatal autopsy	1. Understand the principles of compassionate communication around parental consent for autopsy 2. Know the relevant information to provide to parents to enable informed choice about perinatal autopsy including the value of autopsy 3. Understand the common barriers to obtaining consent for autopsy and be able to discuss possible solutions
2. Autopsy and placental examination	To describe the perinatal autopsy procedure and demonstrate the process of placental examination	1. Understand the procedure of perinatal autopsy and the appearance of a baby after an autopsy examination 2. Be able to perform clinical examination of the placenta and cord 3. Understand the clinical information required by the pathologist to undertake a high quality autopsy examination
3. Investigation of perinatal deaths	To demonstrate the approach to investigation of stillbirths according to the PSANZ Guidelines	1. Understand the timing, type and the reasons for the core investigations for stillbirths 2. Understand extra targeted investigations for stillbirths 3. Understand the role of alternative non-invasive investigations where permission for autopsy is not obtained
4. Examination of babies who die in the perinatal period	To demonstrate how to undertake a detailed clinical examination of stillbirths and neonatal deaths including measurements and clinical photographs	1. Use the recommended checklist to examine a baby following perinatal death 2. Measure a baby and plot these measurements on centile charts 3. Perform recommended standardised clinical photographs
5. Audit and classification of perinatal deaths	To provide an understanding of the process of perinatal mortality audit and the importance of documentation and classification as to causes and contributing factors for stillbirths and neonatal deaths	1. Know the process for high quality perinatal mortality audit 2. Understand the purpose and benefits of classifications using the PSANZ perinatal mortality classifications for stillbirths and neonatal deaths. 3. Understand how to apply the classification system
6. Psychological and social aspects of perinatal bereavement	To provide an understanding of how to provide appropriate psychological and social support for families after a stillbirth and neonatal death	1. Understand parental responses after experiencing perinatal death 2. Know the factors considered to be important for bereaved parents which will influence their experience and outcome 3. Be able to provide appropriate information to parents concerning birth options, special circumstances, creating memories, hospital stay, funeral arrangements and aftercare
Formative assessment	To consolidate key learning objectives for each of the IMPROVE stations	1. Have increased confidence in applying the principles learned to real life clinical scenarios around stillbirths and neonatal deaths 2. Identify areas for additional learning 3. Understand where to find help to address additional learning requirements

### Study setting

The programme was taught across all seven states and territories in Australia, utilising local state/territory coordinators (see acknowledgements section) all of whom are members of PSANZ. Workshops were also conducted in Hanoi (Vietnam), Suva (Fiji), and Amsterdam (The Netherlands). For these international workshops, local champions were identified by the site organisers to assist in the organisation of the programme. This coordination included: arrangement of interpreters in Vietnam, arrangement of teaching facilities, dissemination of information regarding the programme and enrolment of attendees. The local champions were also encouraged to identify attendees who could become educators for each of the stations for future delivery of the programme.

As this study was part of a clinical improvement programme to implement national guidelines and conforms to the standards established by the National Health and Medical Research Council for ethical quality review, [15] ethical approval was not sought.

### Participants

Between June 2012 and May 2014, 26 IMPROVE workshops were conducted across all seven Australian States and Territories. Five workshops were conducted in non-tertiary (secondary) level hospitals, one at the PSANZ Conference and the remainder in tertiary level hospitals. Locations for the workshops were chosen due to local interest in the IMPROVE programme, i.e. a request from that hospital.

Four workshops were held in Hanoi (Vietnam), Suva (Fiji) and Amsterdam (The Netherlands) between October 2013 and May 2015 with 136 attendees. The workshops in Hanoi and Amsterdam were held in conjunction with International Stillbirth Alliance Meetings and the workshops in Suva (Fiji) in conjunction with the Regional Pathology Symposium in Fiji Meetings. Pre- and post-programme evaluation and satisfaction data are available from 76 participants who attended the workshops conducted in Fiji and The Netherlands (100%). Due to translation issues with the forms a complete evaluation of attendees from Vietnam was not available. Locations for the international workshops were chosen based on interest from the meeting/conference organisers.

### Data collection

Details on the participants’ professions were collected at each workshop. Pre- and post-programme knowledge and confidence for content in each station was assessed as well as satisfaction with the programme (as described below).

Participants completed a 16-item questionnaire before and after each workshop to assess their knowledge and confidence relating to the objectives of the six learning stations (Table 1). Knowledge and confidence were assessed using three items for each of stations one to four, and two items were used for each of stations five and six. These items are rated on a 5-point Likert scale (1 = Strongly Disagree to 5 = Strongly Agree). Total confidence and knowledge scores for stations one to four range from 3 to 15 and for stations five and six 2–10, with a total range of 16–80 for overall confidence and knowledge. Participants are rated as confident and knowledgeable for each station if they agree or strongly agree with each item for that station. The proportion of participants who were confident or knowledgeable in perinatal mortality audit was calculated as those agreeing or strongly agreeing with at least 100% of all items in the questionnaire.

Seven items were completed using a 5-point Likert scale (1 = Poor, 3 = Average, and 5 = Excellent), at the end of the workshop to determine satisfaction with: presentation, content, relevance to work, ease of understanding, opportunity for hands on practice/interaction, tutor support and feedback, and overall rating. Participants were categorised as satisfied in that domain if they gave a 4 or 5 for that item. Open-ended questions allowed participants to provide more in-depth feedback about the programme on aspects they found most and least useful, and any suggestions for improvements or overall comments.

### Statistical analyses

To address the first aim of this study (to determine the effectiveness of the IMPROVE programme) data were analysed in a number of ways to test our apriori hypothesis that participants would be more knowledgeable and confident after attending the IMPROVE workshop. We examined effectiveness at the level of the individual item, the station and overall. To aid in interpretation of findings and relevance for policy and decision makers, we examined the proportion of participants who were knowledgeable and confident. To further investigate the effectiveness of the program, it was decided to examine the change in scores for each station and overall as this information can be used to examine the size of the change. Data were assessed for normality to determine appropriate tests to analyse the data. McNemar’s tests were used to examine differences in knowledge and confidence for individual items and each station from pre- and post-IMPROVE workshop. McNemar’s tests were used as the proportions of participants who were knowledgeable and confident at pre- and post-IMPROVE workshop were paired nominal data. Differences in confidence and knowledge, for each station and overall, between doctors and midwives attending the Australian workshops were assessed using Wilcoxon rank-sum tests as the data were non-normally distributed continuous variables. Change between pre- and post-workshop scores were normally distributed so they were assessed using paired *t*-tests, separately for Australian and International participants. Logistic regression models were used to evaluate whether group size (4–6 participants vs 7–9 participants) was associated with the likelihood of being knowledgeable and confident in each station and overall at the end of the workshop, for Australian workshops only. These models were adjusted simultaneously for level of hospital (tertiary or secondary), participants’ profession (doctor, midwife, nurse, other, not stated), educator, and pre-workshop confidence and knowledge. Logistic regression was used as a number of explanatory variables were examined to determine the outcome (knowledgeable and confident in each station or overall) which was a dichotomous variable. To address the second aim of this study (satisfaction with the IMPROVE programme) level of participant satisfaction with each domain and overall was described. The effect of group size (4–6 participants vs 7–9 participants) on satisfaction was assessed using chi-squared tests as the data were categorical. Analyses were conducted separately for Australian and International workshops to address the third aim of this study (determine the suitability of this programme for international use in middle and high income countries). All quantitative analyses were conducted in Stata version 13 (Stata Corporation, College Station TX). Statistical significance was set at *p* < 0.05.

A review of the open-ended satisfaction questions from the Australian workshops was performed with responses grouped into overarching themes. These themes were developed directly from the data and focused on the structure of the workshop and on the translation of learning/knowledge into clinical practice changes. Data from the international workshops were examined to determine if these same themes applied and whether any additional themes emerged and to assess suitability of the IMPROVE programme for use in middle and high income countries.

## Results

A total of 891 people attended the programme with 758 participants providing data for this study (85.1%). For the Australian workshops, most participants were midwives (*n* = 418/758 [55%]), with the remaining participants consisting of 182 (24%) doctors, 54 (7%) nurses, 50 (7%) who listed their profession as other; and 54 (7%) who did not disclose their profession. Thirty-eight doctors, 17 midwives, three nurses, seven other health care professionals and 11 people who did not disclose their profession attended the international workshops. Group size for each Australian workshop ranged from four to nine participants with a median group size of six participants.

Table 2 shows the proportion of participants who were knowledgeable and confident in each item and station pre- and post-workshop. The proportion of Australian participants who were confident and knowledgeable in items relating to audit and classification of perinatal death was approximately one-third that of international participants before the workshops. Other proportions were similar across the other stations. The highest proportion of participants from both groups were confident and knowledgeable in station 6 (Psychological and Social Aspects of Perinatal Bereavement). For four items (2.3, 3.3, 4.1 and 5.2), less than 25% of Australian participants were confident before the workshop while the international participants only had one item (4.1) with this proportion of pre-workshop confidence and knowledge. Over 95% of Australian participants were confident and knowledgeable in 10 items after the workshop with this level of confidence only reported for seven items by international participants. The proportion of Australian and international participants who were knowledgeable and confident increased from pre- to post- workshop for all items and stations.

**Table 2:** Proportion of participants who were knowledgeable and confident for each item and station at pre- and post-IMPROVE workshop (Australian workshops conducted from May 2012 to October 2014; International workshops conducted from November 2013 to May 2015)

	Australian (*N* = 622)		International (*N* = 65)	
Item	Pre (%)	Post (%)	Pre (%)	Post (%)
Station 1 - Communicating Perinatal autopsy				
1.1	56.4	99.2^a^	54.1	100.0^a^
1.2	70.6	99.4^a^	49.2	96.7^a^
1.3	28.2	95.2^a^	49.2	95.1^a^
Overall Station 1 Results	19.9	94.7^a^	23.0	95.1^a^
Station 2 - Autopsy and placenta examination				
2.1	51.9	99.0^a^	36.1	93.4^a^
2.2	53.7	88.4^a^	47.5	90.2^a^
2.3	24.3	92.0^a^	39.3	82.0^a^
Overall Station 2 Results	13.7	84.6^a^	19.7	77.0^a^
Station 3 – Investigation of Perinatal Deaths				
3.1	44.7	96.4^a^	31.1	87.9^a^
3.2	28.8	97.4^a^	31.1	88.5^a^
3.3	21.5	92.6^a^	31.1	91.8^a^
Overall Station 3 Results	15.0	90.8^a^	19.7	82.0^a^
Station 4 – Examination of baby				
4.1	19.5	95.7^a^	23.0	95.1^a^
4.2	55.1	96.0^a^	59.0	88.5^b^
4.3	32.6	89.7^a^	31.1	93.4^a^
Overall Station 4 Results	12.9	87.3^a^	13.1	85.2^a^
Station 5 – Audit and Classification				
5.1	25.7	97.1^a^	41.0	95.1^a^
5.2	14.1	86.7^a^	36.1	93.4^a^
Overall Station 5 Results	10.9	86.0^a^	29.5	91.8^a^
Station 6 - Bereavement				
6.1	56.6	94.5^a^	60.7	96.7^a^
6.2	55.8	96.8^a^	52.5	98.4^a^
Overall Station 6 Results	43.6	92.6^a^	45.9	95.1^a^

^a^ *p* <0.001; ^b^ *p* < 0.01 for difference between pre- and post-workshop as assessed by McNemar’s test

**Fig. 1:**
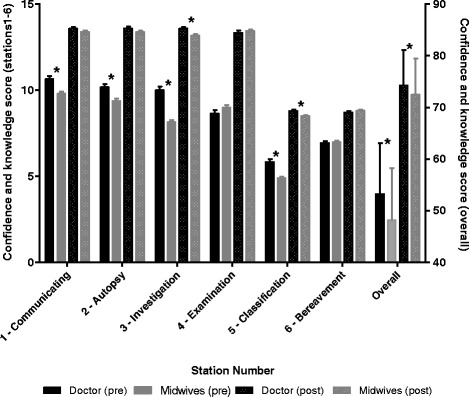
Mean (standard deviation) pre- and post-workshop confidence and knowledge levels for each IMPROVE station, and overall, for doctors (*N* = 160) and midwives (*N* = 361), Australian workshops (conducted from May 2012 to October 2014). * *p* < 0.05 for difference between doctors and midwives as assessed by Wilcoxon rank-sum tests

Table 3 shows pre- and post-workshop confidence and knowledge levels. Participants’ confidence and knowledge significantly increased in each station and overall for participants who attended the Australian and international workshops. Figure 1 shows the pre- and post-workshop confidence and knowledge levels for doctors and midwives who attended the Australian workshops. Doctors reported more confidence and knowledge than midwives before the workshop in stations one, two, three and five and overall. At the completion of the workshop, confidence and knowledge was similar among doctors and midwives/nurses for most stations, however doctors still reported higher scores for stations three and five, and overall.

**Table 3:** Participants’ mean (standard deviation) knowledge of and confidence in each station at pre- and post- IMPROVE workshop, and mean change (95% confidence interval) from pre- to post-IMPROVE workshop (Australian workshops conducted from May 2012 to October 2014; International workshops conducted from November 2013 to May 2015)

	Australian workshops (*N* = 622)				International workshops (*N* = 65)			
Station	Pre-	Post-	Change	Significance^a^	Pre-	Post-	Change	Significance^a^
	Mean (SD)	Mean (SD)	Mean (95% CI)		Mean (SD)	Mean (SD)	Mean (95% CI)	
1 - Communicating Perinatal autopsy								
	10.0 (2.4)	13.4 (1.4)	3.5 (3.3, 3.6)	<0.001	10.3 (2.3)	13.0 (1.4)	2.7 (2.2, 3.2)	<0.001
2 - Autopsy and placenta examination								
	9.3 (2.6)	13.2 (1.7)	4.0 (3.8, 4.1)	<0.001	9.5 (2.6)	12.7 (1.8)	3.2 (2.6, 3.9)	<0.001
3 – Investigation of Perinatal Deaths								
	8.5 (2.7)	13.2 (1. 6)	4.7 (4.5, 4.9)	<0.001	8.8 (2.7)	12.6 (1.7)	3.8 (3.2, 4.4)	<0.001
4 – Examination of baby								
	8. 7 (2.7)	13.3 (1.6)	4.6 (4.4, 4.8)	<0.001	9.3 (2.5)	13.1 (1.7)	3.7 (3.0, 4.5)	<0.001
5 – Audit and Classification								
	5.1 (1.9)	8.5 (1.1)	3.4 (3.3, 3.6)	<0.001	6.2 (2.0)	8.7 (1.1)	2.5 (1.9, 3.1)	<0.001
6 - Bereavement								
	7.0 (1.7)	8.8 (1.1)	1.8 (1.7, 2.0)	<0.001	7.2 (1.6)	8.9 (1.0)	1.7 (1.3, 2.1)	<0.001
Overall	48.4 (10. 7)	72.4 (7.2)	24.0 (23.2, 24.8)	<0.001	51.1 (10.4)	68.5 (6.8)	17.4 (14.6, 20.2)	<0.001

^a^Significance assessed using paired *t*-tests, *SD* standard deviation, *CI* confidence interval

Compared to a group size of 4–6 participants, groups with 7–9 participants have increased odds ratios for post-workshop confidence in stations 1–5 and overall, but the results were not statistically significant (Station 1 OR 2.7 [95% CI 0.9–8.2]; Station 2 OR 1.8 [95% CI 0.9–3.7]; Station 3 OR 2.4 [95% CI 1.0–6.0]; Station 4 OR 1.7 [95% CI 0.8–3.5]; Station 5 OR 1.7 [95% CI 0.9–3.1]; Station 6 OR 0.8 [95% CI 0.4–1.6]; Overall OR 1.5 [95% CI 0.9–2.3]).

Table 4 shows that participants in both settings reported a high level of satisfaction with all domains of the IMPROVE workshops and overall. Group size was not related to satisfaction for any domains or the overall workshop, *p* > 0.1.

**Table 4:** Proportion of participants satisfied with domains of the IMPROVE workshops (Australian workshops conducted from May 2012 to October 2014; International workshops conducted from November 2013 to May 2015)

	Australia (*N* = 622)	International (*N* = 65)
Domain	Satisfaction^a^ (%)	Satisfaction^a^ (%)
Presentation	94	97
Content	95	96
Relevance to work	95	91
Ease of understanding	94	96
Opportunity for hands on practice/interaction	94	73
Tutor support and feedback	94	91
Overall	94	97

^a^Participants were categorised as satisfied in that domain if they scored a 4 or 5 for that item (where 5 = excellent)

Comments from participants reflecting the themes that emerged from the analyses of the open-ended comments are shown in Table 5.

**Table 5:** Participant experience of the IMPROVE programme (Australian workshops conducted from May 2012 to October 2014; International workshops conducted from November 2013 to May 2015)

Item/Theme	Australia	International
Most useful aspects of IMPROVE		
SCORPIO	*“I found it very helpful to have a mix of disciplines (multidisciplinary) in each group, visual + interactive learning, short stations, relevant information” [Midwife, New South Wales]*	*“The small group format worked well for me; practically oriented”* [Doctor, Fiji]
Learning/understanding	*“Learning about what happens during an autopsy. Now confident I could explain this to patients” [Doctor, South Australia]* *“[The] importance of accurate examination of the baby; relevant investigations” [Midwife, South Australia]*	*“The placental swabbing because I have been hearing midwives mentioning it but have no idea how it is done”* [Other, Fiji]
Translation into clinical practice	*“Being able to learn about aspects of perinatal loss that will assist with my care of women, more understanding of the autopsy process- from consent to autopsy. Better understanding of investigations.” [Midwife, New South Wales]*	*“All the stations were very useful and improved my understanding of perinatal mortality reviews and will really help my current practice”* [Doctor, Fiji]
Increased confidence	*“Now feel more confident in knowledge, reacting to autopsy/clinical photography” [Midwife, Queensland]*	*“I feel confident in providing appropriate information to parents”* [Participant, Fiji]
Least useful aspects of IMPROVE		
Audit and classification of perinatal death	*“Institutional perinatal mortality audit and classification, difficult to understand, not well presented, too much information for 30 min.” [Midwife, South Australia]*	*“I least enjoyed session five, but that is just because it is not relevant to my profession”* [Social worker, Amsterdam]
Time	*“Too short - would have loved to have longer time at each station!!” [Midwife, New South Wales]*	*“More time for interaction; 30 min is too short”* [Doctor, Amsterdam]
Suggestions/overall comments		
More IMPROVE	*“This workshop should be made mandatory to all staff exposed to such clinical situations.” [Midwife, New South Wales]*	*“If we could have more of these workshops so we all speak the same language of knowledge and practice”* [Midwife, Fiji] *“Let's try and get a collaboration on a Dutch version!”* [Doctor, Amsterdam]
Satisfaction	*“Very useful day. Very worthwhile. I will be able to make some changes in our practice and educate our staff. Helped me understand much better why we do things and sometimes, how.” [Midwife, South Australia]*	*“Inspiring session”* [Doctor, Amsterdam] *“Thank you so much for this golden opportunity. I really appreciate this.”* [Nurse, Fiji]
Educators	*“Facilitators fantastic-very knowledgeable” [Midwife, New South Wales]*	*“Wonderful presenters”* [Doctor, Fiji]]

Three key themes emerged related to the most useful aspects of the IMPROVE programme: SCORPIO model, learning/understanding, and translation of content into practice. Comments were positive about the structure of the workshop as a mode of clinical education. Participants stated they enjoyed the rotation of small multi-disciplinary groups through the varied stations; this interactive and rotating structure reportedly kept participants engaged, and provided them with an opportunity to discuss the content and their experiences amongst a multi-disciplinary group. IMPROVE was described by participants as being informative and providing them with a better understanding of the processes around perinatal mortality. In particular, participants most commonly reported Station 2 (Autopsy and Placental Examination) as valuable to their work. Many participants were previously unaware of the autopsy process; they reported that learning about this process gave them the confidence to discuss it with affected parents. Participants also reported Stations 3 (Investigations of Perinatal Deaths) and 4 (Examination of babies who die in the perinatal period) to be informative as these provided them with an understanding of the additional and alternative investigations to autopsy. Many comments reflect participants’ intent to apply their acquired knowledge to clinical practice in the care of patients.

The aspect participants most frequently reported as least useful was Station 5 (Audit and classification of perinatal deaths). They also felt that insufficient time was allocated to each station. Reasons for the lack of perceived usefulness of station 5 included: it being less interactive than other stations, not directly applicable to their work, too much information to be adequately addressed in the time given, lack of confidence regarding the content, and finding this station was not well presented. Participants felt that 30 min per station could be extended by 10–15 min as they felt 30 min was not adequate time to address the content. Many wanted additional time per station (particularly for stations 1 and 6) for further discussion amongst the group and to ask questions; they also felt there was enough content to extend the workshop to a full day.

Comments from the participants at the international workshops showed the same themes as those from the Australian workshops. However, there were some additional comments about needing to ensure modification of the content to make it applicable for all participants: *“For the international version of IMPROVE do the investigation workshop more international as well as the classification workshop”* [Doctor, Amsterdam]. One other theme emerged from the international workshops related to opportunity for hands on practice: *“the workshop would greatly improve if they were more hands-on”* [Doctor, Fiji].

Of particular note was that participants from middle-income countries felt that the material was relevant to their workplace and their goals for healthcare provision, despite the fact that the programme was designed in a high-income country and based on the recommended guidelines for that country. Only minor changes were considered necessary for the content for the grief and bereavement station to be culturally relevant.

## Discussion

This study demonstrates that a SCOPRIO style educational programme improves knowledge and confidence of professionals in the management of a perinatal death in both high and middle-income setting. Participants also reported a high level of satisfaction with the programme, as reflected in both qualitative and quantitative data. Our results are consistent with those of Allen and Jeffery, where a SCORPIO style educational course in the low-income setting of Nepal was successful in increasing care providers’ knowledge and competence in newborn care, [13] however IMPROVE still needs to be evaluated in low income countries which have the greater burden of perinatal deaths.

In Australia, midwives reported lower confidence and knowledge than doctors in most of the stations. Midwives also reported lower confidence in talking with parents about autopsy, consistent with the findings of a survey in the UK [8]. It is likely that autopsy counselling is seen as a role performed exclusively by medical officers in Australia and the UK, however evidence would suggest that this is not necessarily imperative, and that other roles may provide benefit. Indeed, in the UK study, a lower proportion of midwives had received training in this area compared to medical officers [8]. Importantly, many of the differences between the professions were not evident at the completion of the workshops. Due to smaller numbers for the international workshops, these comparisons were not investigated.

The station covering audit and classification of perinatal deaths was seen as least useful by a number of participants. This may be related to the fact that few staff members are involved in the classification process as this is done at different hospital or health department levels in the different states, territories and countries. Despite its perceived limited relevance to some participants, this station showed the largest increase in confidence and knowledge. This indicates a potential to have broader involvement of staff in institutional audit, classification and feedback which is a key component of quality improvement in healthcare and can reduce deaths [16]. More knowledge and insight on different causes of death could also increase confidence and knowledge of other stations as these are intertwined.

While the SCORPIO methodology recommends a group size of five participants, [14] we showed that increasing the size of the group (up to nine participants) did not affect learning outcomes, as measured by the likelihood of being confident and knowledgeable in each station and overall, or satisfaction with the workshop. This has implications for programme delivery because larger workshops are more cost effective to run. In fact there were some improved outcomes with larger groups which may be related to more discussion and sharing in each station. While it is conceivable that participants in workshops with larger group sizes may have a different experience, e.g. less opportunity for hands on participation, there was no difference in satisfaction ratings when examined across group size, and similar themes emerged from the open ended responses regardless of group size.

A large number of health care professionals provided their services as educators for IMPROVE workshops. Without covering the costs of educators funds are still required for printing of material, administration time etc. and needs to be considered as integral to maintaining the programme. There are also significant challenges for language translation of materials both from a time but also cost perspective. Without the support of Translators without Borders the Vietnam workshop would have been extremely expensive. These professionals are not reimbursed for their time and appear motivated by altruistic reasons such as a desire to improve patient care through teaching others and passing on their skills and knowledge, consistent with the findings of a previous review [17]. IMPROVE uses a multidisciplinary approach. Participants are able to accumulate continuing professional development points, while addressing a public health issue, which are all themes that have been identified with transforming continuing medical education in the USA and UK [18].

Similar outcomes related to confidence and knowledge were observed regardless of setting, with participants reporting high levels of satisfaction with the IMPROVE programme. Formal statistical comparisons across settings were not conducted due to the imbalance in numbers between the Australian and international settings, and those attending the international workshops may have different knowledge and background as they may have had a key interest in the area by the nature of the programme being run attached to the International Stillbirth Alliance conferences in the Netherlands and Vietnam. Some of the international workshops were conducted in hotels/conference venues where it was not possible to have placentas available for examination. This may reflect the lower satisfaction for the item related to opportunity for hands on practice and the additional themes that emerged from the post-workshop comments in these settings. Nevertheless, these findings demonstrate the utility of IMPROVE in settings other than Australia and it is encouraging that the programme will be made available to healthcare professionals who are caring for babies and families around the time of perinatal death in the Pacific region and other areas as facilitated by ISA. Local champions in Hanoi, Vietnam and Suva, Fiji have been identified and local workshops are due to be run. This programme however, needs to be evaluated in low income settings where local practices may include verbal autopsy due to limited access to perinatal autopsy and placental pathology. Adaptation to local content should be made as required while maintaining ongoing fidelity with the programme. It is important to have close contact with the local team to identify local differences to ensure that local participants have a high degree of satisfaction with the course.

Strengths of this study were the large number of participants, workshops were conducted in all parts of Australia in both tertiary and secondary hospitals and in high and middle-income countries. However, we were limited in that we did not collect more detailed information on the profession of participants e.g. types of medical officers by specialty, such as obstetrician, neonatologist or pathologist, or length of time working in perinatal healthcare.

Limitations of the study were that data has only been collected immediately after the workshop, and that the tool was designed specifically for IMPROVE and has not been validated. It is possible that due to the issue of multiple significance testing our findings appear more favourable as we categorise participants as knowledgeable and confident to aid in interpretation of the findings and assessed differences and changes at the station level and not at the level of the individual item. However, the score for each individual item improved from pre- to post-workshop (data not shown) and also the proportion of participants who were knowledgeable and confident in each item improved form pre- to post-workshop. While quantitative data shows that knowledge and confidence were increased in the short term, no assessment has been made to determine whether this increase in knowledge persists and has resulted in changes in clinical practice, such as increased numbers of autopsies, placental examinations and investigations. Further, while participants were generally satisfied with the programme and intended to take their knowledge and confidence back to their clinical work, external factors such as environmental and organisational barriers will affect participants’ capacity to implement their learnings [19]. A careful, theory-driven approach would enable a more nuanced understanding of the factors underlying behaviour, and may therefore enhance the long-term effectiveness of the programme [16, 20].

## Conclusions

The IMPROVE programme is effective at increasing confidence and knowledge of participants in managing perinatal deaths. The programme has been well received in Australia and in three different international settings and is now being made available through ISA. Future research is required to determine whether the immediate positive outcomes of IMPROVE are sustained and extends to changes in clinical practice and improvement in parents’ experiences with care.

## Abbreviations

CI: Confidence interval
IMPROVE: IMproving Perinatal mortality Review and Outcomes Via Education
ISA: International stillbirth alliance
OR: Odds ratio
PSANZ: Perinatal society of Australia and New Zealand
SCORPIO: Structured, clinical, objective, referenced, problem-orientated, integrated and organised
SD: Standard deviation
